# Cholera outbreak caused by drug resistant *Vibrio cholerae* serogroup O1 biotype ElTor serotype Ogawa in Nepal; a cross-sectional study

**DOI:** 10.1186/s13756-016-0122-7

**Published:** 2016-06-04

**Authors:** Pappu Kumar Gupta, Narayan Dutt Pant, Ramkrishna Bhandari, Padma Shrestha

**Affiliations:** Department of microbiology, Kathmandu college of science and technology, Kathmandu, Nepal; Department of microbiology, Grande international hospital, Dhapasi Kathmandu, Nepal; National public health laboratory, Kathmandu, Nepal

**Keywords:** *Vibrio cholerae*, El Tor, Ogawa, Cholera, Epidemic, Multidrug resistance

## Abstract

**Background:**

Cholera is a major cause of mortality and morbidity in underdeveloped countries including Nepal. Recently drug resistance in *Vibrio cholerae* has become a serious problem mainly in developing countries. The main objectives of our study were to investigate the occurrence of *Vibrio cholerae* in stool samples from patients with watery diarrhea and to determine the antimicrobial susceptibility patterns of *V. cholerae* isolates.

**Methods:**

A total of 116 stool samples from patients suffering from watery diarrhea during July to December 2012 were obtained from outbreak areas from all over Nepal. Alkaline peptone water and thiosulphate citrate bile salt sucrose agar (TCBS) were used to isolate the *Vibrio cholerae*. The isolates were identified with the help of colony morphology, Gram’s staining, conventional biochemical testing, serotyping and biotyping. Antimicrobial susceptibility testing was performed by determining the minimum inhibitory concentration (MIC) by agar dilution method.

**Results:**

*Vibrio cholerae* was isolated from 26.72 % of total samples. All isolated *Vibrio cholerae* were confirmed to be *Vibrio cholerae* serogoup O1 biotype El Tor and serotype Ogawa. All isolates were resistant to ampicillin and cotrimoxazole. Twenty nine isolates were resistant toward two different classes of antibiotics, one strain was resistant to three different classes of antibiotics and one strain was resistant to four different classes of antibiotics. According to the definition of the multidrug resistant bacteria; 6.45 % of the strains of *Vibrio cholerae* were found to be multidrug resistant.

**Conclusions:**

Cholera due to multidrug resistant *Vibrio cholerae* is also possible in Nepal. According to the antimicrobial susceptibility pattern of *Vibrio cholerae* in our study we recommend to use any antibiotics among tetracycline, doxycycline, levofloxacin, azithromycin, chloramphenicol and ciprofloxacin for preliminary treatment of cholera in Nepal.

## Background

*Vibrio cholerae* is one of the most notorious enteric pathogens responsible for many cholera outbreaks [1]. Once commonly detected throughout the world, the infection is now mainly confined to the under-developed countries, where the conditions of drinking water, sanitation and hygiene are not well maintained. It is endemic in Africa, South Asia, and Latin America. Cholera outbreaks usually occur when the drinking water and public sanitation systems are disrupted by natural disasters like earthquakes, tsunamis, volcanic eruptions, landslides and floods or due to crowding like in war displaced refugee camps [2]. Globally, there are an estimated 3–5 millions cholera cases and 100,000–120,000 deaths every year. Commonly, lack of prompt, proper treatment leads to shock within 6–12 h followed by death occurring between 18 h and several days [3].

Cholera is endemic in Nepal [4] and causes massive morbidity and mortality in every monsoon in both urban and rural areas. In Nepal every year 30,000–40,000 people die due to diarrheal diseases, majority of the deaths occurring due to cholera [5]. In recent years, large cholera outbreaks have occurred in western regions of Nepal. In Jajarkot in 2009, within a three weeks period of a cholera outbreak 3,000 people were affected and more than 80 died. The outbreak was associated with poor hygiene and use of contaminated water. The lack of proper medical facilities has further increased the morbidity and mortality in such places [4]. In Nepalgunj, an outbreak of cholera affected more than 1500 people with eight deaths in late July and August 2010 [6]. Outbreaks from other parts of Nepal have also been reported [7–10]. Although several pathogens may be responsible for causing acute diarrhea in humans, it is important to investigate *Vibrio cholerae* as the causative agent, particularly in resource poor settings because it can be fatal, causing death within several hours and it is highly contagious with a propensity to cause epidemics or pandemics [9].

Recently, drug resistance in *Vibrio cholerae* has become a serious problem mainly in developing countries and reports of drug resistance to different antibiotics (including ampicillin, tetracycline, streptomycin, kanamycin, trimethoprim, sulphonamides and gentamicin) have appeared from many cholera-endemic countries [11]. Further cholera cases due to multidrug resistant *Vibrio cholerae* have been reported from all around the globe [12]. Multidrug resistant (MDR) bacteria are the bacteria showing resistance towards three or more than three different classes of antibiotics [13].

In Nepal, only a few studies have been done targeting the identification of drug resistance in *Vibrio cholerae* and limited information is available about the MDR cholera. In this study we investigated the occurrence of *V. cholerae* in stool samples from patients with watery diarrhea and determined the antimicrobial susceptibility patterns of *V. cholerae* isolates. This study will be helpful for the clinicians to start the timely preliminary treatment, by choosing the effective antibiotics for the treatment of the cholera.

## Methods

To determine the involvement of *V. cholerae* in causing watery diarrhea and to determine their antimicrobial susceptibility patterns, a community based study was conducted during rainy season of 2012. For this a total of 116 stool samples {Kathmandu (*n* = 65), Doti (*n* = 30), Bajhang (*n* = 15), Saptari (*n* = 6)} from all patients suffering from watery diarrhea during July to December 2012 were received at National public health laboratory, Kathmandu from outbreak areas from all over Nepal. The samples were transported within 24 h of collection using alkaline peptone water. The stool samples inoculated in enrichment media (alkaline peptone water) were incubated at 37 °C for overnight and were subcultured on selective media, TCBS. The characteristic yellow colored (sucrose fermenting) colonies grown on TCBS, after 24 h of aerobic incubation at 37 °C were subjected to biochemical testing [14] and serotyping using specific antisera (Denka Seiken, Tokyo, Japan) following manufactures instructions. The biotyping was performed with the help of Voges Proskauer test (by using methyl red Voges Proskauer media), chicken erythrocyte agglutination test and susceptibility to polymixin B (50 iu). The El Tor biotype is Voges Proskauer test positive, shows agglutination and is resistant to polymixin B [14]. Antimicrobial susceptibility testing to ampicillin, cotrimoxazole, tetracycline, doxycycline, chloramphenicol, ciprofloxacin, levofloxacin and azithromycin was performed by determining the minimum inhibitory concentration (MIC) by agar dilution method as suggested by Andrews [15]. The different dilutions used for ampicillin, tetracycline, doxycycline, chloramphenicol, ciprofloxacin, levofloxacin and azithromycin were 0.5 μg/ml, 1 μg/ml, 2 μg/ml, 4 μg/ml, 8 μg/ml, 16 μg/ml, 32 μg/ml, 64 μg/ml, 128 μg/ml, 256 μg/ml. For cotrimoxazole the different dilution used were 0.5/9.5 μg/ml, 1/19 μg/ml, 2/38 μg/ml, 4/76 μg/ml, 8/152 μg/ml, 16/204 μg/ml, 32/408 μg/ml. For the interpretation of the antimicrobial susceptibility, MIC breakpoints as suggested by CLSI document M45 guidelines were used [16].

Strains showing resistance towards ≥3 different classes of drugs were considered as multidrug resistant [13]. The data obtained were entered into MS excel and analyzed using SPSS version 11.0. Chi square test was used and *p*-value < 0.05 was taken as significant.

## Results

Of total 116 samples received, 31 (26.72 %) samples were found to be positive for *V. cholerae* among which 13 (41.93 %) were from male patients and 18 (58.07 %) were from female patients. The cholera agent was identified as *Vibrio cholerae* serogroup O1 biotype El Tor and serotype Ogawa. Most of *V. cholerae* (70.97 %) were isolated from patients from Kathmandu valley.

### Age wise distribution of cholera cases

All age groups were found to be affected by cholera, among which the people belonging to age group of 20–39 years were affected most and the people belonging to age group of above 60 years were affected least (*P* < 0.05) (Table 1).

**Table 1:** Age wise distribution of cholera cases

Age group (years)	Number (%)
≤19	8 (25.81)
20–39	15 (48.89)
40–59	6 (19.35)
≥60	2 (6.45)
Total	31 (100)

### Location wise distribution of cholera cases

33.84 % of the samples from Kathmandu and 20 % of the samples from Bajhang were found to be positive for *V. cholerae* (Table 2).

**Table 2:** Location wise distribution of cholera cases

Location	Total samples	Positive samples (%)
Kathmandu	65	22 (33.85 %)
Doti	30	5 (16.67 %)
Bajhang	15	3 (20 %)
Saptari	6	1 (16.67 %)

### Antibiotic susceptibility patterns of *Vibrio cholerae* toward different commonly used antibiotics

All 31 isolates were resistant to ampicillin and cotrimoxazole. Similarly, all of the 31 isolates were sensitive to tetracycline, doxycycline, levofloxacin and azithromycin, while 30/31 (96.77 %) of the isolates were sensitive to chloramphenicol and 29/31 (93.55 %) of the isolates were sensitive to ciprofloxacin (Table 3).

**Table 3:** Antibiotic susceptibility patterns of *Vibrio cholerae* toward different commonly used antibiotics

Antimicrobials	No. of strains	Susceptibility patterns					
		Sensitive (%)	MIC (μg/ml)	MIC breakpoints for sensitive strains (μg/ml)	Resistant (%)	MIC (μg/ml)	MIC breakpoints for resistant strains (μg/ml)
Ampicillin	31	0	-	≤8	100	64	≥32
Cotrimoxazole	31	0	-	≤2/38	100	8/152	≥4/76
Tetracycline	31	100	2	≤4	0	-	≥16
Doxycycline	31	100	2	≤4	0	-	≥16
Chloramphenicol	31	96.77	8	≤8	3.23	32	≥32
Ciprofloxacin	31	93.55	0.5	≤1	6.45	4	≥4
Levofloxacin	31	100	0.5	≤2	0	-	≥8
Azithromycin	31	100	1	≤2	0	-	≥8

### Distribution of resistant strains of *Vibrio cholerae* against different drugs

Twenty-nine isolates were resistant toward two different classes of antibiotics, 1 strain was resistant to three different classes of antibiotics and one strain was resistant to four different classes of antibiotics.

### Drug resistant phenotype of *V. cholerae* isolates

The resistance of the strains to particular combination of antibiotics was used to differenciate different drug resistant phenotypes of *V. cholerae*. Twenty-nine of the isolates were resistant to ampicillin (Amp)-cotrimoxazole (TS), 1 isolate was resistant to ampicillin (Amp)-cotrimoxazole (TS)-ciprofloxacin (CIP), 1 isolate was resistant to ampicillin (Amp)-cotrimoxazole (TS)-ciprofloxacin (CIP)-chloramphenicol (C) (Table 4).

**Table 4:** Drug resistant phenotype of *V. cholerae* isolates

Antibiotic resistant patterns	No. of strains
AMP-TS	29
AMP-TS-CIP	1
AMP-TS-CIP-C	1

## Discussion

In the present study, the incidence of cholera among the cases of watery diarrhea was found to be 26.72 % which is similar to the findings of Tamang et al. (31 %) [7] and Karki et al. (27.1 %) [17]. All the strains isolated in our study were *Vibrio cholerae* serogroup O1 serotype Ogawa and biotype El Tor and the finding is consistent with the studies done in other parts of Nepal [2, 8, 10, 17]. However in contrast to our study, there was a total serotype conversion to Inaba in 2005 and 2006 while in 2007, all three serotypes {Ogawa (64 %), Inaba (35 %) and Hikojima (1 %)} were isolated [18]. Such a serotype shifting is a common phenomenon in *V. cholerae* [19–21].

The contaminated drinking water may have contributed for highest cholera cases in Kathmandu valley in our study [22]. The drinking water of Kathmandu is highly contaminated and is responsible for many other water related infections also.

In our study 41.93 % of the *Vibrio cholerae* isolates were isolated from male patients and 58.07 % of the strains were isolated from female patients. Similar results were also reported by Pun et al. who isolated 57 % of the strains from females and 43 % from males [9]. We found the most affected age group to be 20–39 years which is in accordance with the results reported by other researchers [8, 9, 17, 23]. The food habits of eating outside the home and consumption of street foods may have contributed to the high incidence of cholera in this age group.

Recently there have been reports of increased drug resistance toward commonly used antibiotics among the strains of *Vibrio cholerae*, causing serious problem in management of the cholera cases [11]. In a study by Karki et al. one hundred percent resistance was observed for cotrimoxazole which supports our findings [17] but in contrast to our study the isolates were found to be highly susceptible to ampicillin [17] and high rates of susceptibility were reported toward tetracycline, ciprofloxacin, and erythromycin [17]. However, in our study all isolates were sensitive to tetracycline, doxycycline, levofloxacin and azithromycin, while most of the isolates were sensitive to chloramphenicol (96.77 %) and ciprofloxacin (93.55 %). As in our study all strains were resistant to cotrimoxazole and ampicillin in the studies done by Shrestha et al. [24] and Das et al. [25]. The majority of *V. cholerae* strains were identified as susceptible to tetracycline (100 %), ciprofloxacin (90.9 %), cefotaxime (81.8 %) and chloramphenicol (90.9 %) by Shrestha et al. [24] and which is in accordance with the results we have reported. However, in contrast to our study, Shah et al*.* [26] showed that 81.8 % of strains were resistant to tetracycline. Bhandari et al. reported all isolates to be sensitive to commonly used antibiotics except nalidixic acid and cotrimoxazole [8]. Similar as in our study, 100 % resistance to ampicillin and 97.8 % susceptibility to ciprofloxacin were reported by Karki and Tiwari [27]. Generally, fluoroquinolones are highly effective for treatment of cholera but recently fluoroquinolone resistant strains of *V. cholerae* have been reported from India [24]. The difference in drug susceptibility of the strains of *Vibrio cholerae* isolated from different places during different periods of time may be due to mutation over time or due to the haphazard uses of different antibiotics in different places or during different periods of time [24]. And the antibiotics for the treatment of the cholera should be selected on the basis of the local antimicrobial susceptibility patterns of the *Vibrio cholerae*.

In our study we found 6.45 % of the strains to be multidrug resistant but Shrestha et al. reported the rate of multidrug resistance to be 100 % [24]. Multidrug resistant *Vibrio cholerae* have been reported from all around the world including Pakistan, Bangladesh, India and Nepal [24]. Changing antibiogram profile of *Vibrio cholerae*, developing MDR strains over years may be attributed to the spontaneous mutation due to indiscriminate use of antibiotics or horizontal transfer of resistance genes [24, 28]. So the use of the antibiotics for the management of the cholera cases should be based on the antibiotic susceptibility patterns of *Vibrio cholerae* in the particular area. Further, the rate of cholera can be controlled to significant level by giving awareness regarding the methods of control of the cholera (like personal hygiene) to the public. As shown in a recent review of the USA Centers for Disease Control and Prevention (CDC) surveillance data over a period of 21 years (1990–2010), patients may get different antibiotics for treatment [29]. But to investigate the potential correlation between laboratory results and the surveillance findings, antimicrobial susceptibility patterns to a combination of antimicrobials were not available [29]. So the antimicrobial susceptibility patterns of the *Vibrio* spp. to different commonly used antibiotics may be helpful.

### Limitation of the study

The main limitation of this study was lack of funding and unavailability of sophisticated laboratory tests (as this research was conducted in low income country), due to which we could not use molecular methods to confirm our results.

## Conclusions

The cholera due to multidrug resistant *Vibrio cholerae* is also possible in Nepal. The local antimicrobial susceptibility patterns of the *Vibrio cholerae* is necessary to start a proper timely treatment of cholera. The antimicrobial therapy must be based on the antimicrobial susceptibility testing report of *Vibrio cholerae*. According to the antimicrobial susceptibility patterns of *Vibrio cholerae* in our study we recommend to use any antibiotics among tetracycline, doxycycline, levofloxacin, azithromycin, chloramphenicol and ciprofloxacin for preliminary treatment of cholera in Nepal.

## Abbreviations

AMP, ampicillin; C, chloramphenicol; CDC, center for disease control and prevention; CIP, ciprofloxacin; CLSI, clinical and laboratory standards institute; MDR, multidrug resistant; MIC, minimum inhibitory concentration; MS, microsoft; SPSS, statistical package for the social sciences; TCBS, thiosulphate citrate bile salt agar; TS, cotrimoxazole; USA, United States of America.
